# A Method to Compute the Schrieffer–Wolff Generator for Analysis of Quantum Memory

**DOI:** 10.3390/e23101260

**Published:** 2021-09-27

**Authors:** Dong-Hwan Kim, Su-Yong Lee, Yonggi Jo, Duk Y. Kim, Zaeill Kim, Taek Jeong

**Affiliations:** Emerging Science and Technology Directorate, Agency for Defense Development, Daejeon 34186, Korea; kiow639@add.re.kr (D.-H.K.); yonggi@add.re.kr (Y.J.); duk0@add.re.kr (D.Y.K.); zkim@add.re.kr (Z.K.)

**Keywords:** quantum illumination, transmon-cavity quantum memory, equivalent circuit, Schrieffer–Wolff transformation

## Abstract

Quantum illumination uses entangled light that consists of signal and idler modes to achieve higher detection rate of a low-reflective object in noisy environments. The best performance of quantum illumination can be achieved by measuring the returned signal mode together with the idler mode. Thus, it is necessary to prepare a quantum memory that can keep the idler mode ideal. To send a signal towards a long-distance target, entangled light in the microwave regime is used. There was a recent demonstration of a microwave quantum memory using microwave cavities coupled with a transmon qubit. We propose an ordering of bosonic operators to efficiently compute the Schrieffer–Wolff transformation generator to analyze the quantum memory. Our proposed method is applicable to a wide class of systems described by bosonic operators whose interaction part represents a definite number of transfer in quanta.

## 1. Introduction

Quantum memories are required to store and retrieve quantum states with high fidelity. To synchronize various events, quantum memories are essential for quantum information networks, including quantum computation [1], quantum communication [2], and quantum illumination [3]. Quantum illumination (QI), a target detection scheme using quantum entangled light with signal and idler modes, has as its objective enhancing the detection rate of a target with low-reflectivity in a highly noisy environment [3]. In QI, the signal mode is sent to the target while the idler mode is retained. Although the noisy environment destroys the entanglement between the signal and idler modes, we can take quantum advantage over the classical limit by jointly measuring the returned signal mode and the idler mode when the signal arrives [4,5,6,7]. During this process, it is highly appreciable to keep the idler mode in an ideal quantum memory. This was investigated using various systems, such as a microwave cavity [8], mechanical oscillators [9], or spin ensembles [10,11].

Here, we focus on quantum memories using microwave cavities that can have high-quality factors and allow continuous-variable quantum information processes. By coupling a microwave cavity to a transmon qubit, it is able to write arbitrary states on the cavity and infer information about the cavity [8,12]. It is based on the cross Kerr effect, where the energy gap of neighboring levels of the cavity (transmon qubit) depends on the excitations of the transmon qubit (cavity). The anharmonicity of the transmon qubit gives rise to the cross Kerr effect through coupling of the qubit and cavity [13,14].

In dealing with such systems, it is crucial to understand how the coupling affects the energy structure. The Schrieffer–Wolff transformation computes this shift in energy structure by using a basis change unitary to remove the coupling [15,16]. For multiple bosonic modes containing nonlinear terms, it is complicated to find the exact generator of the unitary for the Schrieffer–Wolff transformation. Here, we propose a systematic approach to find the generator and compute the energy corrections induced by this transformation. An ordering of operators, which we call computational ordering, greatly simplifies the commutation structure of operators making it suitable in calculating the Schrieffer–Wolff transformation generator.

## 2. Equivalent Circuit Analysis of a Quantum Memory

**Figure 1:**
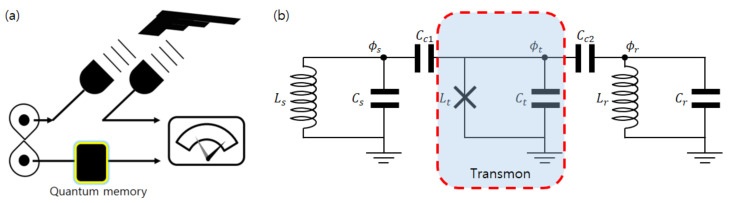
(**a**) Concept of quantum illumination with a quantum memory. (**b**) Equivalent circuit model of quantum memory under consideration. Transmon is coupled to two LC oscillators via capacitors. Symbols with *L* represent inductances, while symbols with *C* represent capacitances. $\phi_{i}$ at each specified node is flux variable used in Appendix A.

The quantum memory demonstrated in Ref. [8] couples two microwave cavities through a transmon qubit. One cavity is used as a memory (storage) to store quantum states and the other cavity is used as a readout port whose response depends on the memory-cavity state through the transmon qubit. Such a system can be described by an equivalent circuit depicted in Figure 1b. Two LC oscillators represent the microwave cavities, while the middle oscillator represents the transmon qubit. The oscillators are labeled as *s*, *t*, and *r* for storage, transmon, and readout, respectively, as in Ref. [8]. The transmon qubit is coupled to both cavities by capacitors. The Hamiltonian describing this system is

(1) $$\frac{\hat{H}}{\hbar }=\sum_{i=s,t,r}\omega_{i}{\hat{a}}_{i}^{†}{\hat{a}}_{i}-\frac{E_{C}}{2\hbar }{\hat{a}}_{t}^{†}{\hat{a}}_{t}^{†}{\hat{a}}_{t}{\hat{a}}_{t}+g_{1}\left({\hat{a}}_{s}^{†}{\hat{a}}_{t}+{\hat{a}}_{s}{\hat{a}}_{t}^{†}\right)+g_{2}\left({\hat{a}}_{t}^{†}{\hat{a}}_{r}+{\hat{a}}_{t}{\hat{a}}_{r}^{†}\right),$$

where ${\hat{a}}_{s}$, ${\hat{a}}_{t}$, ${\hat{a}}_{r}$ are bosonic annihilation operators corresponding to each oscillator mode. A detailed derivation of this Hamiltonian and expressions of $\omega_{s},\omega_{t},\ldots$ in terms of $L_{i}$, $C_{i}$, $C_{c1}$, $C_{c2}$(i=s,t,r) are given in Appendix A. We assume that the system is in the dispersive regime, where the couplings $g_{1}$ and $g_{2}$ are much smaller than the detunings $|\omega_{s}-\omega_{t}|$ and $|\omega_{t}-\omega_{r}|$.

The elimination of capacitive couplings in Equation (1) gives rise to cross Kerr effects among each cavity and the transmon. This is done by diagonalizing the Hamiltonian. There are two ways in achieving this; namely, second-order perturbation and the Schrieffer–Wolff transformation [16]. These methods were previously applied to systems of transmon qubits coupled with LC oscillators in evaluating the energy structure [13,14]. For the Schrieffer–Wolff transformation, one must find an operator $\hat{S}$, the Schrieffer–Wolff generator, which is an off-diagonal operator satisfying a given commutator equation. It is complicated to determine the operator $\hat{S}$ and compute various commutators to obtain the second-order energy corrections. Thus, we introduce a method that simplifies the computation and apply it to Equation (1). After the computation, we truncate the transmon qubit to the lowest two levels to obtain a Jaynes–Cummings-like Hamiltonian [13]. Truncation of the transmon qubit should be done after diagonalization since virtual excitations of the transmon need to be considered.

### 2.1. Computational Ordering for Schrieffer–Wolff Transformation

We propose an ordering of bosonic operators, which gives direct computation of the Schrieffer–Wolff generator and second-order energy corrections. For a short recall of the Schrieffer–Wolff transformation, let $\hat{H}$ be the Hamiltonian in interest. We separate the Hamiltonian into diagonal and off-diagonal parts, $\hat{H_{0}}$, $\hat{V}$ respectively, so $\hat{H}={\hat{H}}_{0}+\hat{V}$. The Schrieffer–Wolff generator $\hat{S}$ is defined as the off-diagonal operator which satisfies $\left[\hat{S},\hat{H_{0}}\right]=-\hat{V}$. Then,

(2) $$e^{\hat{S}}\hat{H}e^{-\hat{S}}={\hat{H}}_{0}+\frac{1}{2}\left[\hat{S},\hat{V}\right]+\ldots .$$

The energy separations of ${\hat{H}}_{0}$ must be larger than $\hat{V}$, so that $\hat{S}$ becomes small and a perturbative approach is applicable [17]. This condition becomes evident when we write down the explicit form of $\hat{S}$ in Equation (5). The second-order energy corrections to ${\hat{H}}_{0}$ are given as the diagonal part of $\frac{1}{2}\left[\hat{S},\hat{V}\right]$.

We consider a system of *N* bosonic modes, where ${\hat{a}}_{i}$ is the annihilation operator of the *i*-th mode and satisfies $\left[{\hat{a}}_{i},{\hat{a}}_{j}^{†}\right]=\delta_{ij}$. We propose an ordering of operators

(3) $${\hat{a}}^{†n}f\left({\hat{a}}^{†}\hat{a}\right){\hat{a}}^{m}$$

to efficiently compute the Schrieffer–Wolff transformation generator and second-order energy corrections to ${\hat{H}}_{0}$. In Equation (3), ${\hat{a}}^{†}\hat{a}=\left({\hat{a}}_{1}^{†}{\hat{a}}_{1},\ldots ,{\hat{a}}_{N}^{†}{\hat{a}}_{N}\right)$, $n=(n_{1},\ldots ,n_{N})$, $m=(m_{1},\ldots ,m_{N})$ are *N* tuples and ${\hat{a}}^{†n}={\hat{a}}_{1}^{†n_{1}}\ldots {\hat{a}}_{N}^{†n_{N}}$, ${\hat{a}}^{m}={\hat{a}}_{1}^{m_{1}}\ldots {\hat{a}}_{N}^{m_{N}}$. To ensure that *f* is unique, we require n, m to have disjoint support, i.e. $n\cdot m:=\left(n_{1}m_{1},\ldots ,n_{N}m_{N}\right)=\left(0,\ldots ,0\right)$. For example, the operator ${\hat{a}}_{1}^{†}{\hat{a}}_{1}^{†}{\hat{a}}_{1}^{†}{\hat{a}}_{1}{\hat{a}}_{1}$ will be written as

$$\begin{array}{cc} {\hat{a}}_{1}^{†}{\hat{a}}_{1}^{†}{\hat{a}}_{1}^{†}{\hat{a}}_{1}{\hat{a}}_{1} & ={\hat{a}}_{1}^{†}\left({\hat{a}}_{1}^{†}{\hat{a}}_{1}{\hat{a}}_{1}^{†}{\hat{a}}_{1}-{\hat{a}}_{1}^{†}{\hat{a}}_{1}\right)={\hat{a}}_{1}^{†}f\left({\hat{a}}^{†}\hat{a}\right),\\ f(x_{1},\ldots ,x_{N}) & =x_{1}^{2}-x_{1}. \end{array}$$

The main motivation of this ordering is that diagonal operators in the Fock basis correspond to functions defined on $N_{0}^{N}$, with $N_{0}=\left\{0,1,2,3,\ldots \right\}$, and functions are in general easier to manipulate than operators. The computational ordering is then equivalent to writing a given operator in terms of number operators as much as possible. Explicit expressions for writing normal-ordered or antinormal-ordered operators in this ordering are given in Appendix B. Note that operators that have n=m=0 are exactly the diagonal operators in the Fock basis.

We write the Hamiltonian $\hat{H}$ in this ordering:

(4) $$\hat{H}=f\left({\hat{a}}^{†}\hat{a}\right)+\sum_{n,m}{\hat{a}}^{†n}g_{nm}\left({\hat{a}}^{†}\hat{a}\right){\hat{a}}^{m}.$$

The sum, here and henceforth, is over all **n**, **m** satisfying n·m=(0,…,0) and **n**, **m** are not both 0. This automatically splits the Hamiltonian into diagonal and off-diagonal parts. The hermitian condition on $\hat{H}$ forces *f* to be real valued and $g_{nm}^{*}=g_{mn}$, $z^{*}$ being the complex conjugate of *z*. The main results are

(5) $$\begin{array}{cc} \hat{S} & =\sum_{n,m}{\hat{a}}^{†n}\frac{g_{nm}\left({\hat{a}}^{†}\hat{a}\right)}{f\left({\hat{a}}^{†}\hat{a}+n\right)-f\left({\hat{a}}^{†}\hat{a}+m\right)}{\hat{a}}^{m}, \end{array}$$

(6) $$\begin{array}{cc} \frac{1}{2}{\left[\hat{S},\hat{V}\right]}_{\left(d\right)} & =\sum_{n,m}\frac{{\left({\hat{a}}^{†}\hat{a}\right)}^{\underline{n}}{\left({\hat{a}}^{†}\hat{a}-n+m\right)}^{\underline{m}}{\left|g_{nm}\left({\hat{a}}^{†}a-n\right)\right|}^{2}}{f\left({\hat{a}}^{†}\hat{a}\right)-f\left({\hat{a}}^{†}\hat{a}-n+m\right)}. \end{array}$$

where $x^{\underline{n}}=x\left(x-1\right)\ldots \left(x-n+1\right)$ is the falling factorial and the falling factorial of tuples is defined element-wise. The subscript ${}_{\left(d\right)}$ means to take the diagonal part, so $\frac{1}{2}{\left[\hat{S},\hat{V}\right]}_{\left(d\right)}$ is the second-order correction to energy. Since $f,g_{nm}$ are essentially functions defined on $N_{0}^{N}$ as noted before, the computation of Equations (5) and (6) is straightforward. In the end, the original Hamiltonian is transformed via the Schrieffer–Wolff transformation as

(7) $$e^{\hat{S}}\hat{H}e^{-\hat{S}}={\hat{H}}_{0}+\frac{1}{2}{\left[\hat{S},\hat{V}\right]}_{\left(d\right)}+\cdots ={\hat{H}}_{0}+{\hat{H}}^{\left(2\right)}+\ldots$$

where the omitted terms are off-diagonal terms of second-order in $\hat{V}$ or diagonal terms of third-order in $\hat{V}$. The superscript ${}^{\left(2\right)}$ indicates that the term is second-order in $\hat{V}$.

To obtain the main results Equations (5) and (6), we need a computational lemma.

**Lemma** **1.** *The commutators of ${\hat{a}}^{†n}$, ${\hat{a}}^{m}$ with $f({\hat{a}}^{†}\hat{a})$ are as follows.*

(8) $$\begin{array}{cc} \left[{\hat{a}}^{†n},f\left({\hat{a}}^{†}\hat{a}\right)\right] & ={\hat{a}}^{†n}\left(f\left({\hat{a}}^{†}\hat{a}\right)-f\left({\hat{a}}^{†}\hat{a}+n\right)\right), \end{array}$$

(9) $$\begin{array}{cc} {\hat{a}}^{m},f\left({\hat{a}}^{†}\hat{a}\right)] & =\left(f\left({\hat{a}}^{†}\hat{a}+m\right)-f\left({\hat{a}}^{†}\hat{a}\right)\right){\hat{a}}^{m}. \end{array}$$

**Proof.**  It suffices to check on number states $|k\rangle =|k_{1},\ldots ,k_{N}\rangle$. One can verify

$$\begin{array}{cc} {}[{\hat{a}}^{†n},f\left({\hat{a}}^{†}\hat{a}\right)]|k\rangle & ={\hat{a}}^{†n}f\left({\hat{a}}^{†}\hat{a}\right)|k\rangle -f\left({\hat{a}}^{†}\hat{a}\right){\hat{a}}^{†n}|k\rangle \\ \; & =f\left(k\right){\hat{a}}^{†n}|k\rangle -{\left(\prod_{i=1}^{N}{\left(k_{i}+1\right)}^{\bar{n_{i}}}\right)}^{1/2}f\left({\hat{a}}^{†}\hat{a}\right)|k+n\rangle \\ \; & ={\left(\prod_{i=1}^{N}{\left(k_{i}+1\right)}^{\bar{n_{i}}}\right)}^{1/2}\left(f\left(k\right)-f\left(k+n\right)\right)|k+n\rangle \\ \; & ={\hat{a}}^{†n}\left(f\left(k\right)-f\left(k+n\right)\right)|k\rangle \\ \; & ={\hat{a}}^{†n}\left(f\left({\hat{a}}^{†}\hat{a}\right)-f\left({\hat{a}}^{†}\hat{a}+n\right)\right)|k\rangle . \end{array}$$

Here, $x^{\bar{n}}=x\left(x+1\right)\ldots \left(x+n-1\right)$ is the rising factorial. The commutator with ${\hat{a}}^{m}$ follows from taking the adjoint. □

Now one can compute the commutator of $\hat{S}$ with ${\hat{H}}_{0}$. Write $\hat{S}$ as

(10) $$\hat{S}=\sum_{n,m}{\hat{a}}^{†n}h_{nm}\left({\hat{a}}^{†}\hat{a}\right){\hat{a}}^{m},$$

with $h_{nm}^{*}=-h_{mn}$ so that $\hat{S}$ is antihermitian. Then, one has

$$\begin{array}{cc} \left[\hat{S},{\hat{H}}_{0}\right] & =\sum_{n,m}\left[{\hat{a}}^{†n}h_{nm}\left({\hat{a}}^{†}\hat{a}\right){\hat{a}}^{m},{\hat{H}}_{0}\right]\\ \; & =\sum_{n,m}{\hat{a}}^{†n}h_{nm}\left({\hat{a}}^{†}\hat{a}\right)\left(f\left({\hat{a}}^{†}\hat{a}+m\right)-f\left({\hat{a}}^{†}\hat{a}+n\right)\right){\hat{a}}^{m}. \end{array}$$

The choice of $\hat{S}$ as in Equation (5) yields $\left[\hat{S},\hat{H_{0}}\right]=-\hat{V}$, i.e., we take $h_{nm}$ as

(11) $$h_{nm}\left({\hat{a}}^{†}\hat{a}\right)=\frac{g_{nm}\left({\hat{a}}^{†}\hat{a}\right)}{f\left({\hat{a}}^{†}\hat{a}+n\right)-f\left({\hat{a}}^{†}\hat{a}+m\right)}.$$

The conditions on *f*, $g_{nm}$ ensure that $h_{nm}^{*}=-h_{mn}$ holds. This is well-defined as long as the diagonal part is nondegenerate, which is true when considering low excitations of transmons.

**Figure 2:**
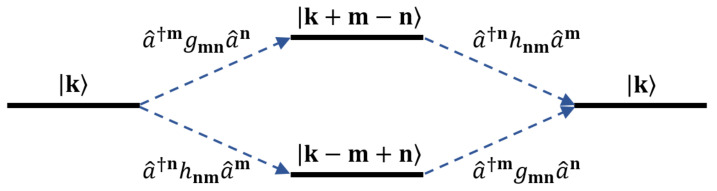
Schematic representation of diagonal terms in the commutator $\frac{1}{2}\left[\hat{S},\hat{V}\right]$. The Fock basis state |k〉 must end up in |k〉 to give a diagonal contribution. For example, in the product $\hat{S}\hat{V}$, the ${\hat{a}}^{†m}g_{mn}\left({\hat{a}}^{†}\hat{a}\right){\hat{a}}^{n}$ term in $\hat{V}$ maps the state |k〉 to |k+m−n〉. The only term in $\hat{S}$ which maps this back to |k〉 is ${\hat{a}}^{†n}h_{nm}\left({\hat{a}}^{†}\hat{a}\right){\hat{a}}^{m}$. This corresponds to the upper-half of the above diagram. The lower-half of the diagram represents the $\hat{V}\hat{S}$ product.

Using the generator $\hat{S}$ defined as Equation (5), we can compute the correction to energies as the diagonal part of $\frac{1}{2}\left[\hat{S},\hat{V}\right]$. The only term in $\hat{V}$ that gives a diagonal contribution with the ${\hat{a}}^{†n}h_{nm}\left({\hat{a}}^{†}\hat{a}\right){\hat{a}}^{m}$ term in $\hat{S}$ is ${\hat{a}}^{†m}g_{mn}\left({\hat{a}}^{†}\hat{a}\right){\hat{a}}^{n}$. A pictorial representation of this statement is given in Figure 2. Hence,

(12) $$\begin{array}{cc} \frac{1}{2}{\left[\hat{S},\hat{V}\right]}_{\left(d\right)} & =\frac{1}{2}\sum_{n,m}\left[{\hat{a}}^{†n}h_{nm}\left({\hat{a}}^{†}\hat{a}\right){\hat{a}}^{m},{\hat{a}}^{†m}g_{mn}\left({\hat{a}}^{†}\hat{a}\right){\hat{a}}^{n}\right] \end{array}$$

(13) $$\begin{array}{c} \begin{array}{cc} \; & =\frac{1}{2}\sum_{n,m}\left\{\frac{{\left({\hat{a}}^{†}\hat{a}\right)}^{\underline{n}}{\left({\hat{a}}^{†}\hat{a}-n+m\right)}^{\underline{m}}{\left|g_{nm}\left({\hat{a}}^{†}a-n\right)\right|}^{2}}{f\left({\hat{a}}^{†}\hat{a}\right)-f\left({\hat{a}}^{†}\hat{a}-n+m\right)}\right.\\ \; & \;+\left.\frac{{\left({\hat{a}}^{†}\hat{a}\right)}^{\underline{m}}{\left({\hat{a}}^{†}\hat{a}+n-m\right)}^{\underline{n}}{\left|g_{nm}\left({\hat{a}}^{†}a-m\right)\right|}^{2}}{f\left({\hat{a}}^{†}\hat{a}\right)-f\left({\hat{a}}^{†}\hat{a}+n-m\right)}\right\} \end{array} \end{array}$$

(14) $$\begin{array}{cc} \; & =\sum_{n,m}\frac{{\left({\hat{a}}^{†}\hat{a}\right)}^{\underline{n}}{\left({\hat{a}}^{†}\hat{a}-n+m\right)}^{\underline{m}}{\left|g_{nm}\left({\hat{a}}^{†}a-n\right)\right|}^{2}}{f\left({\hat{a}}^{†}\hat{a}\right)-f\left({\hat{a}}^{†}\hat{a}-n+m\right)}. \end{array}$$

The calculation of the commutator can be done by using Lemma 1 and results in Appendix B. Note that the summand in Equation (13) is symmetric under change of n, m, which leads to Equation (14). This result is equivalent to nondegenerate second-order perturbation energy correction,

(15) $$E_{k}^{\left(2\right)}=\sum_{k^{\prime }\neq k}\frac{{\left|\langle k^{\prime }\left|\hat{V}\right|k\rangle \right|}^{2}}{E_{k}-E_{k^{\prime }}},$$

computed in our proposed ordering.

In most cases, interaction terms are of form ${\hat{a}}_{i}^{†}g_{ij}\left({\hat{a}}^{†}\hat{a}\right){\hat{a}}_{j}$, which represent a single transfer of quantum excitations. If we restrict the interaction to only these terms, our main results Equations (5) and (6) are simplified to

(16) $$\begin{array}{cc} \hat{S} & =\sum_{i\neq j}{\hat{a}}_{i}^{†}\frac{g_{ij}\left({\hat{a}}^{†}\hat{a}\right)}{f\left({\hat{a}}^{†}\hat{a}+e_{i}\right)-f\left({\hat{a}}^{†}\hat{a}+e_{j}\right)}{\hat{a}}_{j}, \end{array}$$

(17) $$\begin{array}{cc} \frac{1}{2}{\left[\hat{S},\hat{V}\right]}_{\left(d\right)} & =\sum_{i\neq j}\frac{{\hat{a}}_{i}^{†}{\hat{a}}_{i}\left({\hat{a}}_{j}^{†}{\hat{a}}_{j}+1\right){\left|g_{ij}\left({\hat{a}}^{†}\hat{a}-e_{i}\right)\right|}^{2}}{f\left({\hat{a}}^{†}\hat{a}\right)-f\left({\hat{a}}^{†}\hat{a}-e_{i}+e_{j}\right)}, \end{array}$$

where $e_{i}$ is the *N* tuple, which has 1 as its *i*-th component, and all other elements are 0 and i,j∈{1,2,…,N}.

### 2.2. Application to Analyzing the Circuit Hamiltonian

We return to the diagonalization of the circuit Hamiltonian Equation (1). To apply the previous formalism, define functions $f,g_{12},g_{23}$ as

(18) $$\begin{array}{cc} f(n,m,\ell ) & :=\omega_{s}n+\omega_{t}m+\omega_{r}\ell -\frac{E_{C}}{2\hbar }m\left(m-1\right), \end{array}$$

(19) $$\begin{array}{cc} g_{12}\left(n,m,\ell \right) & =g_{21}\left(n,m,\ell \right):=g_{1}, \end{array}$$

(20) $$\begin{array}{cc} g_{23}\left(n,m,\ell \right) & =g_{32}\left(n,m,\ell \right):=g_{2}. \end{array}$$

These functions give a full description of the Hamiltonian Equation (1). n,m,ℓ correspond to ${\hat{a}}_{s}^{†}{\hat{a}}_{s}$, ${\hat{a}}_{t}^{†}{\hat{a}}_{t}$, ${\hat{a}}_{r}^{†}{\hat{a}}_{r}$, respectively. The second-order energy corrections can be directly computed by our main result Equation (17).

(21) $$\begin{array}{c} \begin{array}{cc} \frac{{\hat{H}}^{\left(2\right)}}{\hbar } & =|g_{1}{|}^{2}\left(\frac{n(m+1)}{f(n,m,\ell )-f(n-1,m+1,\ell )}+\frac{m(n+1)}{f(n,m,\ell )-f(n+1,m-1,\ell )}\right)\\ \; & \;+|g_{2}{|}^{2}\left(\frac{m(\ell +1)}{f(n,m,\ell )-f(n,m-1,\ell +1)}+\frac{\ell (m+1)}{f(n,m,\ell )-f(n,m+1,\ell -1)}\right) \end{array} \end{array}$$

(22) $$\begin{array}{c} \begin{array}{cc} \; & =|g_{1}{|}^{2}\left(\frac{{\hat{a}}_{s}^{†}{\hat{a}}_{s}\left({\hat{a}}_{t}^{†}{\hat{a}}_{t}+1\right)}{\Delta_{st}+{\hat{a}}_{t}^{†}{\hat{a}}_{t}E_{C}/\hbar }-\frac{{\hat{a}}_{t}^{†}{\hat{a}}_{t}\left({\hat{a}}_{s}^{†}{\hat{a}}_{s}+1\right)}{\Delta_{st}+\left({\hat{a}}_{t}^{†}{\hat{a}}_{t}-1\right)E_{C}/\hbar }\right)\\ \; & \;+|g_{2}{|}^{2}\left(\frac{{\hat{a}}_{r}^{†}{\hat{a}}_{r}\left({\hat{a}}_{t}^{†}{\hat{a}}_{t}+1\right)}{\Delta_{rt}+{\hat{a}}_{t}^{†}{\hat{a}}_{t}E_{C}/\hbar }-\frac{{\hat{a}}_{t}^{†}{\hat{a}}_{t}\left({\hat{a}}_{r}^{†}{\hat{a}}_{r}+1\right)}{\Delta_{rt}+\left({\hat{a}}_{t}^{†}{\hat{a}}_{t}-1\right)E_{C}/\hbar }\right), \end{array} \end{array}$$

with $\Delta_{st}:=\omega_{s}-\omega_{t}$, $\Delta_{rt}:=\omega_{r}-\omega_{t}$. To read off shifts in frequency, cross Kerr coefficients, and anharmonicities, we must put Equation (22) in normal order:

(23) $$\frac{{\hat{H}}^{\left(2\right)}}{\hbar }=\delta_{s}{\hat{a}}_{s}^{†}{\hat{a}}_{s}+\delta_{t}{\hat{a}}_{t}^{†}{\hat{a}}_{t}+\delta_{r}{\hat{a}}_{r}^{†}{\hat{a}}_{r}+\frac{\delta_{K}}{2\hbar }{\hat{a}}_{t}^{†}{\hat{a}}_{t}^{†}{\hat{a}}_{t}{\hat{a}}_{t}+\chi_{st}{\hat{a}}_{s}^{†}{\hat{a}}_{s}{\hat{a}}_{t}^{†}{\hat{a}}_{t}+\chi_{rt}{\hat{a}}_{r}^{†}{\hat{a}}_{r}{\hat{a}}_{t}^{†}{\hat{a}}_{t}+\ldots .$$

Using the result from Equation (A16), the shifts are given as

(24) $$\begin{array}{c} \begin{array}{cc} \frac{{\hat{H}}^{\left(2\right)}}{\hbar } & =\frac{|g_{1}{|}^{2}}{\Delta_{st}}{\hat{a}}_{s}^{†}{\hat{a}}_{s}+\frac{|g_{2}{|}^{2}}{\Delta_{rt}}{\hat{a}}_{r}^{†}{\hat{a}}_{r}+\sum_{k=1}^{\infty }\frac{{\left(-1\right)}^{k}}{E_{C}}\left(\frac{|g_{1}{|}^{2}}{{\left(\Delta_{st}/E_{C}\right)}^{\bar{k}}}+\frac{|g_{2}{|}^{2}}{{\left(\Delta_{rt}/E_{C}\right)}^{\bar{k}}}\right){\hat{a}}_{t}^{†k}{\hat{a}}_{t}^{k}\\ \; & \;+\sum_{k=1}^{\infty }\frac{{\left(-1\right)}^{k}{\left|g_{1}\right|}^{2}\left(k+1\right)}{E_{C}{\left(\Delta_{st}/E_{C}\right)}^{\bar{k+1}}}{\hat{a}}_{s}^{†}{\hat{a}}_{t}^{†k}{\hat{a}}_{t}^{k}{\hat{a}}_{s}+\sum_{k=1}^{\infty }\frac{{\left(-1\right)}^{k}{\left|g_{2}\right|}^{2}\left(k+1\right)}{E_{C}{\left(\Delta_{rt}/E_{C}\right)}^{\bar{k+1}}}{\hat{a}}_{r}^{†}{\hat{a}}_{t}^{†k}{\hat{a}}_{t}^{k}{\hat{a}}_{r}, \end{array} \end{array}$$

where $x^{\bar{n}}=x\left(x+1\right)\ldots \left(x+n-1\right)$ is the rising factorial and factors of *ℏ* were omitted in the right-hand side for simplicity. Restoring these factors are done by replacing $E_{C}$ with $E_{C}/\hbar$. The shifts in physical quantities are found by simply reading off the coefficients of Equation (24):

(25) $$\begin{array}{cc} \delta_{s} & =\frac{|g_{1}{|}^{2}}{\Delta_{st}},\;\delta_{r}=\frac{|g_{2}{|}^{2}}{\Delta_{rt}},\;\delta_{t}=-\frac{|g_{1}{|}^{2}}{\Delta_{st}}-\frac{|g_{2}{|}^{2}}{\Delta_{rt}}, \end{array}$$

(26) $$\begin{array}{cc} \delta_{K} & =\frac{2|g_{1}{|}^{2}E_{C}}{\Delta_{st}\left(\Delta_{st}+E_{C}/\hbar \right)}+\frac{2|g_{2}{|}^{2}E_{C}}{\Delta_{rt}\left(\Delta_{rt}+E_{C}/\hbar \right)}, \end{array}$$

(27) $$\begin{array}{cc} \chi_{st} & =-\frac{2|g_{1}{|}^{2}E_{C}/\hbar }{\Delta_{st}\left(\Delta_{st}+E_{C}/\hbar \right)},\;\chi_{rt}=-\frac{2|g_{2}{|}^{2}E_{C}/\hbar }{\Delta_{rt}\left(\Delta_{rt}+E_{C}/\hbar \right)}. \end{array}$$

Hence, the total transformed Hamiltonian can be written as

(28) $$\begin{array}{cc} e^{\hat{S}}\frac{\hat{H}}{\hbar } & e^{-\hat{S}}=\frac{1}{\hbar }\left({\hat{H}}_{0}+{\hat{H}}^{\left(2\right)}+\ldots \right)\\ \; & =\sum_{i=s,t,r}\left(\omega_{i}+\delta_{i}\right){\hat{a}}_{i}^{†}{\hat{a}}_{i}-\frac{E_{C}-\delta_{K}}{2\hbar }{\hat{a}}_{t}^{†}{\hat{a}}_{t}^{†}{\hat{a}}_{t}{\hat{a}}_{t}+\chi_{st}{\hat{a}}_{s}^{†}{\hat{a}}_{s}{\hat{a}}_{t}^{†}{\hat{a}}_{t}+\chi_{rt}{\hat{a}}_{r}^{†}{\hat{a}}_{r}{\hat{a}}_{t}^{†}{\hat{a}}_{t}+\ldots . \end{array}$$

This extends the results using Bogoliubov approach to diagonalize the Hamiltonian of a coupled single LC oscillator and transmon [18] in the sense that the frequency shift of the transmon qubit is the sum of contributions from coupling to each LC oscillator. Such a system is described by a Hamiltonian

(29) $$\hat{H}=\hbar \omega_{1}{\hat{a}}^{†}\hat{a}+\hbar \omega_{2}{\hat{b}}^{†}\hat{b}-\frac{E_{C}}{2}{\hat{b}}^{†}{\hat{b}}^{†}\hat{b}\hat{b}+\hbar g\left({\hat{a}}^{†}\hat{b}+\hat{a}{\hat{b}}^{†}\right).$$

Elimination of the $\hbar g({\hat{a}}^{†}\hat{b}+\hat{a}{\hat{b}}^{†})$ term gives rise to cross Kerr coefficient between $\hat{a},\hat{b}$,

(30) $$\chi =-\frac{{2|g|}^{2}E_{C}/\hbar }{\Delta (\Delta +E_{C}/\hbar )},$$

where $\Delta :=\omega_{1}-\omega_{2}$, which highly resembles the results in Equation (27). Note that there is a sign difference in the definition of Δ compared with that of Ref. [18].

To obtain a form similar to that given in Refs. [13,14], we truncate the transmon Hilbert space to the first two levels. Such truncation is done by replacing ${\hat{a}}_{t}^{†}{\hat{a}}_{t}$ with $(\sigma_{z}+1)/2$ in Equation (22). The result is

(31) $$\begin{array}{c} \begin{array}{cc} \frac{{\hat{H}}^{\left(2\right)}}{\hbar } & =\frac{|g_{1}{|}^{2}}{\Delta_{st}+E_{C}/\hbar }{\hat{a}}_{s}^{†}{\hat{a}}_{s}-\left(\frac{|g_{1}{|}^{2}}{\Delta_{st}}+\frac{|g_{2}{|}^{2}}{\Delta_{rt}}\right)\frac{\sigma_{z}}{2}+\frac{|g_{2}{|}^{2}}{\Delta_{rt}+E_{C}/\hbar }{\hat{a}}_{r}^{†}{\hat{a}}_{r}\\ \; & \;-\frac{|g_{1}{|}^{2}E_{C}/\hbar }{\Delta_{st}\left(\Delta_{st}+E_{C}/\hbar \right)}{\hat{a}}_{s}^{†}{\hat{a}}_{s}\sigma_{z}-\frac{|g_{2}{|}^{2}E_{C}/\hbar }{\Delta_{rt}\left(\Delta_{rt}+E_{C}/\hbar \right)}{\hat{a}}_{r}^{†}{\hat{a}}_{r}\sigma_{z} \end{array} \end{array}$$

up to an overall constant. Again, the contributions from each oscillator-transmon coupling stated in Ref. [13] are added independently. The frequency shifts of LC oscillators seem to be different compared with Equation (25), but this is due to a subtle difference of physical interpretation. The coefficients $\delta_{s}$, $\delta_{r}$ in Equation (25) are the frequency shifts when the transmon is in the ground state, while the coefficients of ${\hat{a}}_{s}^{†}{\hat{a}}_{s}$, ${\hat{a}}_{r}^{†}{\hat{a}}_{r}$ in Equation (31) are the average of the frequency shifts when the transmon is in the ground state and excited state. With the ${\hat{a}}_{s}^{†}{\hat{a}}_{s}\sigma_{z}$, ${\hat{a}}_{r}^{†}{\hat{a}}_{r}\sigma_{z}$ terms in consideration, both Equations (22) and (31) give the same energy spectrum when considering up to the first excitation of the transmon.

## 3. Discussion

We proposed an ordering of bosonic operators to efficiently compute the Schrieffer–Wolff transformation generator and energy corrections. This formalism was applied to a system with a transmon coupled to two different LC oscillators to model a quantum memory and readout device demonstrated in Ref. [8]. We solved the normal ordering problem for an operator that appears in the second-order energy correction, so that shifts in physical parameters such as frequency, anharmonicity, and cross Kerr coefficients can be directly read off from the normal-ordered form.

Our proposed method can be directly applied to systems consisting of LC circuits coupled with multiple transmons, and even to systems that have nonlinear couplings provided that the couplings represent a definite number of excitations or de-excitations in the Fock basis. It is possible to generalize this method to incorporate fermionic operators in this formalism, which can be used to reproduce the results of the original application of Schrieffer–Wolff transformation to the Anderson impurity model as in Appendix C. With such a general method, one can analyze a wide range of time-independent systems.

Quantum illumination, an example of quantum information technology, uses entangled light to achieve higher detection rate of a target with low-reflectivity. The idler mode, a part of the entangled light, should be stored in a quantum memory for ideal operation. Our method was used to analyze a demonstrated quantum memory and can be used to analyze other systems operating in various quantum technologies. For further research, it is required to find methods to store and release the propagating idler mode efficiently [19], leading to applications of quantum memories to quantum illumination.

## Data Availability

Data is contained within the article.

## Appendix A. Derivation of Hamiltonian from Circuit QED

In this section, we follow the quantization method of Ref. [20] and obtain various physical quantities, such as the normal mode frequency of each oscillator and the couplings of modes in terms of circuit parameters $L_{i},C_{i},C_{c1},C_{c2}\left(i=s,t,r\right)$. Let $\phi_{i}$ be the flux variable at node i(i=s,t,r) as labeled in Figure 1b. The corresponding (linear) Lagrangian for this system is

(A1) $$L=\frac{1}{2}\left(C_{s}{\dot{\phi_{s}}}^{2}+C_{t}{\dot{\phi_{t}}}^{2}+C_{r}{\dot{\phi_{r}}}^{2}+C_{c1}{\left(\dot{\phi_{s}}-\dot{\phi_{t}}\right)}^{2}+C_{c2}{\left(\dot{\phi_{t}}-\dot{\phi_{r}}\right)}^{2}\right)-\frac{1}{2}\left(\frac{\phi_{s}^{2}}{L_{s}}+\frac{\phi_{t}^{2}}{L_{t}}+\frac{\phi_{r}^{2}}{L_{r}}\right)$$

The conjugate variables are $q_{i}:=\partial L/\partial \dot{\phi_{i}}$ and the Hamiltonian H is

(A2) $$\begin{array}{c} \begin{array}{cc} H & =\frac{1}{2{\bar{C}}^{3}}\left[\begin{array}{ccc} q_{s} & q_{t} & q_{r} \end{array}\right]\left[\begin{array}{ccc} {\bar{C}}_{s}^{2} & C_{c1}\left(C_{r}+C_{c2}\right) & C_{c1}C_{c2}\\ C_{c1}\left(C_{r}+C_{c2}\right) & {\bar{C}}_{t}^{2} & C_{c2}\left(C_{s}+C_{c1}\right)\\ C_{c1}C_{c2} & C_{c2}\left(C_{s}+C_{c1}\right) & {\bar{C}}_{r}^{2} \end{array}\right]\left[\begin{array}{c} q_{s}\\ q_{t}\\ q_{r} \end{array}\right]\\ & \;+\frac{1}{2}\left[\begin{array}{ccc} \phi_{s} & \phi_{t} & \phi_{r} \end{array}\right]\left[\begin{array}{ccc} L_{s}^{-1} & \; & \\ & L_{t}^{-1} & \\ & \; & L_{r}^{-1} \end{array}\right]\left[\begin{array}{c} \phi_{s}\\ \phi_{t}\\ \phi_{r} \end{array}\right], \end{array} \end{array}$$

(A3) $$\begin{array}{c} \begin{array}{cc} {\bar{C}}^{3} & :=C_{s}C_{t}C_{r}+C_{s}C_{t}C_{c2}+C_{s}C_{r}C_{c1}+C_{s}C_{r}C_{c2}+C_{t}C_{r}C_{c1}\\ \; & \;+C_{s}C_{c1}C_{c2}+C_{t}C_{c1}C_{c2}+C_{r}C_{c1}C_{c2}, \end{array} \end{array}$$

(A4) $$\begin{array}{cc} {\bar{C}}_{s}^{2} & :=C_{t}C_{r}+C_{t}C_{c2}+C_{r}C_{c1}+C_{r}C_{c2}+C_{c1}C_{c2}, \end{array}$$

(A5) $$\begin{array}{cc} {\bar{C}}_{t}^{2} & :=\left(C_{s}+C_{c1}\right)\left(C_{r}+C_{c2}\right), \end{array}$$

(A6) $$\begin{array}{cc} {\bar{C}}_{r}^{2} & :=C_{s}C_{t}+C_{s}C_{c1}+C_{t}C_{c1}+C_{s}C_{c2}+C_{c1}C_{c2}. \end{array}$$

Elevating $q_{i},\phi_{i}$ to canonical operators ${\hat{q}}_{i},{\hat{\phi }}_{i}$ with $\left[{\hat{\phi }}_{i},{\hat{q}}_{j}\right]=i\hbar \delta_{ij}$ and defining creation, annihilation operators as usual gives

(A7) $$\begin{array}{c} \begin{array}{cc} \hat{H} & =\sum_{i=s,t,r}\hbar \omega_{i}{\hat{a}}_{i}^{†}{\hat{a}}_{i}\\ \; & \;-\hbar g_{1}\left({\hat{a}}_{s}^{†}-{\hat{a}}_{s}\right)\left({\hat{a}}_{t}^{†}-{\hat{a}}_{t}\right)-\hbar g_{2}\left({\hat{a}}_{t}^{†}-{\hat{a}}_{t}\right)\left({\hat{a}}_{r}^{†}-{\hat{a}}_{r}\right)-\hbar g_{3}\left({\hat{a}}_{s}^{†}-{\hat{a}}_{s}\right)\left({\hat{a}}_{r}^{†}-{\hat{a}}_{r}\right), \end{array} \end{array}$$

(A8) $$\begin{array}{cc} {\hat{q}}_{i} & :=i{\left(\frac{\hbar^{2}{\bar{C}}^{3}}{4L_{i}{\bar{C}}_{i}^{2}}\right)}^{1/4}\left({\hat{a}}_{i}^{†}-{\hat{a}}_{i}\right),\;{\hat{\phi }}_{i}:={\left(\frac{\hbar^{2}L_{i}{\bar{C}}_{i}^{2}}{4{\bar{C}}^{3}}\right)}^{1/4}\left({\hat{a}}_{i}^{†}+{\hat{a}}_{i}\right),\;\omega_{i}^{-2}:=L_{i}{\bar{C}}^{3}/{\bar{C}}_{i}^{2}, \end{array}$$

(A9) $$\begin{array}{cc} g_{1} & :=\frac{C_{c1}\left(C_{r}+C_{c2}\right)}{4{\left(L_{s}L_{t}{\bar{C}}_{s}^{2}{\bar{C}}_{t}^{2}{\bar{C}}^{6}\right)}^{1/4}},\;g_{2}:=\frac{C_{c2}\left(C_{s}+C_{c1}\right)}{4{\left(L_{t}L_{r}{\bar{C}}_{t}^{2}{\bar{C}}_{r}^{2}{\bar{C}}^{6}\right)}^{1/4}},\;g_{3}:=\frac{C_{c1}C_{c2}}{4{\left(L_{s}L_{r}{\bar{C}}_{s}^{2}{\bar{C}}_{r}^{2}{\bar{C}}^{6}\right)}^{1/4}}. \end{array}$$

Note that in the weak coupling limit $\left(C_{c1},C_{c2}\ll C_{s},C_{t},C_{r}\right)$, the eigen frequencies and couplings simplify to

(A10) $$\omega_{i}^{-2}≃L_{i}C_{i},\;g_{1}≃\frac{C_{c1}}{4{\left(L_{s}L_{t}C_{s}^{3}C_{t}^{3}\right)}^{1/4}},\;g_{2}≃\frac{C_{c2}}{4{\left(L_{t}L_{r}C_{t}^{3}C_{r}^{3}\right)}^{1/4}},\;g_{3}≃\frac{4g_{1}g_{2}}{\omega_{t}}.$$

The coupling between ${\hat{q}}_{s},{\hat{q}}_{r}$ is off-diagonal of second-order, and hence, give energy corrections of third- and higher-order. Our analysis concerns up to second-order energy corrections, so we dropped this term. If one wants to consider higher-order corrections, then consideration of this coupling is necessary.

The nonlinearity of the transmon is introduced by replacing ${\hat{\phi }}_{t}^{2}/2L_{t}$ with $-\frac{\Phi_{0}^{2}}{L_{t}}\cos\left(\frac{{\hat{\phi }}_{t}}{\Phi_{0}}\right)$, where $\Phi_{0}=\hbar /2e$ is the flux quantum. Expanding the cosine series up to fourth-order yields

(A11) $$\begin{array}{c} \begin{array}{cc} \frac{\hat{H}}{\hbar } & =\omega_{s}{\hat{a}}_{s}^{†}{\hat{a}}_{s}+\left(\omega_{t}-\frac{E_{C}}{\hbar }\right){\hat{a}}_{t}^{†}{\hat{a}}_{t}+\omega_{r}{\hat{a}}_{r}^{†}{\hat{a}}_{r}\\ \; & \;+g_{1}\left({\hat{a}}_{s}^{†}{\hat{a}}_{t}+{\hat{a}}_{s}{\hat{a}}_{t}^{†}\right)+g_{2}\left({\hat{a}}_{t}^{†}{\hat{a}}_{r}+{\hat{a}}_{t}{\hat{a}}_{r}^{†}\right)-\frac{E_{C}}{2\hbar }{\hat{a}}_{t}^{†}{\hat{a}}_{t}^{†}{\hat{a}}_{t}{\hat{a}}_{t}, \end{array} \end{array}$$

where $E_{C}=e^{2}{\bar{C}}_{t}^{2}/2{\bar{C}}^{3}$ is the charging energy of the transmon, which is small compared with $\hbar \omega_{t}$ in the transmon regime [18]. The correction to $\omega_{t}$ is ignored, while it can be easily recovered in the final results by just replacing $\omega_{t}$ with $\omega_{t}-E_{C}/\hbar$. We applied the rotating wave approximation to remove nonresonant terms such as ${\hat{a}}_{s}^{†}{\hat{a}}_{t}^{†},{\hat{a}}_{t}^{†}{\hat{a}}_{t}^{†}{\hat{a}}_{t}^{†}{\hat{a}}_{t}$ which represent the creation or destruction of two or more quanta.

## Appendix B. Computational Ordering of Normal-Ordered and Antinormal-Ordered Operators

In this section, we give explicit formulas of writing normal-ordered and antinormal-ordered operators in our proposed computational ordering. They extensively use Stirling numbers of the first kind s(n,k), which are the matrix elements of the basis change of monomials $x^{n}$ and falling factorials $x^{\underline{n}}$ [21] (p. 824).

(A12) $$x^{\underline{n}}=\sum_{k=0}^{n}s\left(n,k\right)x^{k}.$$

The relation of rising factorials and monomials is similar, only differing in sign.

(A13) $$x^{\bar{n}}=\sum_{k=0}^{n}{\left(-1\right)}^{n-k}s\left(n,k\right)x^{k}.$$

Since the matrix elements of our computational ordered operators and normal-, antinormal-ordered operators in the Fock basis involve monomials, falling factorials, rising factorials, respectively, it is obvious that Stirling numbers will appear. The results are stated for a single bosonic operator $\hat{a}$. Extension to several bosonic operators is trivial.

(A14) $$\begin{array}{cc} {\hat{a}}^{†n}{\hat{a}}^{m} & =\left\{\begin{array}{ccc} {\hat{a}}^{†n-m}{\left({\hat{a}}^{†}\hat{a}\right)}^{\underline{m}} & ={\hat{a}}^{†n-m}\sum_{k=0}^{m}s\left(m,k\right){\left({\hat{a}}^{†}\hat{a}\right)}^{k} & \left(n\geq m\right)\\ {\left({\hat{a}}^{†}\hat{a}\right)}^{\underline{n}}\;{\hat{a}}^{m-n} & =\sum_{k=0}^{n}s\left(n,k\right){\left({\hat{a}}^{†}\hat{a}\right)}^{k}{\hat{a}}^{m-n} & \left(n\leq m\right) \end{array}\right., \end{array}$$

(A15) $$\begin{array}{cc} {\hat{a}}^{m}{\hat{a}}^{†n} & =\left\{\begin{array}{ccc} {\hat{a}}^{†n-m}{\left({\hat{a}}^{†}\hat{a}+n\right)}^{\underline{m}} & ={\hat{a}}^{†n-m}\sum_{k=0}^{m}s\left(m,k\right){\left({\hat{a}}^{†}\hat{a}+n\right)}^{k} & \left(n\geq m\right)\\ {\left({\hat{a}}^{†}\hat{a}+m\right)}^{\underline{n}}\;{\hat{a}}^{m-n} & =\sum_{k=0}^{n}s\left(n,k\right){\left({\hat{a}}^{†}\hat{a}+m\right)}^{k}{\hat{a}}^{m-n} & \left(n\leq m\right) \end{array}\right.. \end{array}$$

To read off anharmonicity and cross Kerr coefficients as in Equation (23), one must know how to convert operators in our ordering into normal order. We end this section with showing that

(A16) $$\frac{1}{{\hat{a}}^{†}\hat{a}+c}=\sum_{k=0}^{\infty }\frac{{\left(-1\right)}^{k}}{c^{\bar{k+1}}}{\hat{a}}^{†k}{\hat{a}}^{k}=\frac{1}{c}-\frac{{\hat{a}}^{†}\hat{a}}{c(c+1)}+\frac{{\hat{a}}^{†}{\hat{a}}^{†}\hat{a}\hat{a}}{c(c+1)(c+2)}-\ldots$$

where *c* is not a nonpositive integer. This is equivalent to finding $\alpha_{k}$ such that

(A17) $$\frac{1}{n+c}=\sum_{k=0}^{n}\alpha_{k}n^{\underline{k}}.$$

Since ${\left(n^{\underline{k}}\right)}_{nk}$ is a lower diagonal matrix, its inverse exists. The inverse matrix is easily shown to be the lower diagonal matrix

(A18) $${\left(\frac{{\left(-1\right)}^{n+k}}{k!(n-k)!}\right)}_{nk}.$$

Then, the coefficients $\alpha_{k}$ become

(A19) $$\alpha_{k}=\sum_{n=0}^{k}\frac{{\left(-1\right)}^{n+k}}{n!(k-n)!}\frac{1}{n+c}=\frac{{\left(-1\right)}^{k}}{k!c}{\;}_{2}F_{1}\left(c,-k;c+1;1\right)=\frac{{\left(-1\right)}^{k}}{c^{\bar{k+1}}}$$

which proves Equation (A16). Here, ${}_{2}F_{1}\left(a,b;c;z\right)$ is the hypergeometric function [21] (p. 556).

## Appendix C. Generalization to Fermionic Operators

In this section, we adapt our formalism to fermionic operators and apply it to the Anderson impurity model. We consider *n* fermionic modes, described by operators $\left\{{\hat{b}}_{i},{\hat{b}}_{j}^{†}\right\}=\delta_{ij}$, $\{{\hat{b}}_{i},{\hat{b}}_{j}\}=0$, $\{{\hat{b}}_{i}^{†},{\hat{b}}_{j}^{†}\}=0$, where ${\hat{b}}_{i}$ is the annihilation operator of the *i*-th mode. We write operators in the following order as in Equation (3),

(A20) $${\hat{b}}_{I}^{†}f\left({\hat{b}}^{†}\hat{b}\right){\hat{b}}_{J},$$

where ${\hat{b}}^{†}\hat{b}=\left({\hat{b}}_{1}^{†}{\hat{b}}_{1},\ldots ,{\hat{b}}_{n}^{†}{\hat{b}}_{n}\right)$ is an *n* tuple and $I=\{i_{1},\ldots ,i_{k}\}$, $J=\{j_{1},\ldots ,j_{\ell }\}$ are disjoint subsets of {1,2,…,n}. The operators are defined as ${\hat{b}}_{I}^{†}={\hat{b}}_{i_{1}}^{†}\ldots {\hat{b}}_{i_{k}}^{†}$, ${\hat{b}}_{J}={\hat{b}}_{j_{1}}^{†}\ldots {\hat{b}}_{j_{\ell }}^{†}$. We take $i_{1}<i_{2}<\cdots <i_{k}$, $j_{1}<j_{2}<\cdots <j_{\ell }$ to fix ordering and require *I*, *J* be disjoint for the ordering to be unique. Since ${\hat{b}}_{i}$ are fermionic operators, *f* is essentially a function defined on ${\left\{0,1\right\}}^{n}$ and if i∈I∪J, then *f* is independent of the *i*-th variable.

We split the Hamiltonian into diagonal and off-diagonal terms,

(A21) $$\hat{H}=f\left({\hat{b}}^{†}\hat{b}\right)+\sum_{I,J}{\hat{b}}_{I}^{†}g_{IJ}\left({\hat{b}}^{†}\hat{b}\right){\hat{b}}_{J}.$$

The sum is over all disjoint subsets *I*, *J* of {1,2,…,n} where both are not the null set. The hermiticity of $\hat{H}$ makes *f* a real valued function and $g_{IJ}^{*}=g_{JI}$. One then computes the commutator of ${\hat{b}}_{I}^{†}$, ${\hat{b}}_{J}$ with $f({\hat{b}}^{†}\hat{b})$:

(A22) $$\begin{array}{cc} \left[{\hat{b}}_{I}^{†},f\left({\hat{b}}^{†}\hat{b}\right)\right] & ={\hat{b}}_{I}^{†}\left\{f\left(I=0\right)-f\left(I=1\right)\right\}, \end{array}$$

(A23) $$\begin{array}{cc} {\hat{b}}_{J},f\left({\hat{b}}^{†}\hat{b}\right)] & =\left\{f\left(J=1\right)-f\left(J=0\right)\right\}{\hat{b}}_{J}. \end{array}$$

Here, f(I=0) is the operator obtained from $f({\hat{b}}^{†}\hat{b})$ by replacing ${\hat{b}}_{i}^{†}{\hat{b}}_{i}$ with 0 for all i∈I and other operators are defined similarly. This can be seen from noting that ${\hat{b}}_{I}^{†}f\left({\hat{b}}^{†}\hat{b}\right)={\hat{b}}_{I}^{†}f\left(I=0\right)$, $f\left({\hat{b}}^{†}\hat{b}\right){\hat{b}}_{I}^{†}={\hat{b}}_{I}^{†}f\left(I=1\right)$ and similar relations hold for ${\hat{b}}_{J}$. Hence the commutator $\left[{\hat{b}}_{I}^{†}h_{IJ}\left({\hat{b}}^{†}\hat{b}\right){\hat{b}}_{J},f\left({\hat{b}}^{†}\hat{b}\right)\right]$ is

(A24) $$\left[{\hat{b}}_{I}^{†}\;h_{IJ}\left({\hat{b}}^{†}\hat{b}\right)\;{\hat{b}}_{J},f\left({\hat{b}}^{†}\hat{b}\right)\right]={\hat{b}}_{I}^{†}\;h_{IJ}\left({\hat{b}}^{†}\hat{b}\right)\left\{f\left(I=0,\;J=1\right)-f\left(I=1\;J=0\right)\right\}\;{\hat{b}}_{J}.$$

Now we take $h_{IJ}\left({\hat{b}}^{†}\hat{b}\right)$ as follows to ensure $\left[\hat{S},{\hat{H}}_{0}\right]=-\hat{V}$:

(A25) $$\begin{array}{cc} h_{IJ}\left({\hat{b}}^{†}\hat{b}\right) & :=\frac{g_{IJ}\left({\hat{b}}^{†}\hat{b}\right)}{f(I=1,J=0)-f(I=0,J=1)}, \end{array}$$

(A26) $$\begin{array}{cc} \hat{S} & :=\sum_{IJ}{\hat{b}}_{I}^{†}\;h_{IJ}\left({\hat{b}}^{†}\hat{b}\right)\;{\hat{b}}_{J}. \end{array}$$

$h_{IJ}$ does not have the terms ${\hat{b}}_{i}^{†}{\hat{b}}_{i}$ if i∈I∪J.

The diagonal contribution of $\frac{1}{2}\left[\hat{S},\hat{V}\right]$ comes from the terms

(A27) $$\begin{array}{c} \begin{array}{cc} {}[{\hat{b}}_{I}^{†} & \;h_{IJ}\left({\hat{b}}^{†}\hat{b}\right)\;{\hat{b}}_{J},{\hat{b}}_{J}^{†}\;g_{JI}\left({\hat{b}}^{†}\hat{b}\right)\;{\hat{b}}_{I}]\\ \; & =\frac{{\left(-1\right)}^{s(|I|)+s(|J|)}{\left|g_{IJ}\left({\hat{b}}^{†}\hat{b}\right)\right|}^{2}}{f(I=1,\;J=0)-f(I=0,\;J=1)}\left\{{\left({\hat{b}}^{†}\hat{b}\right)}_{I}{\left(1-{\hat{b}}^{†}\hat{b}\right)}_{J}-{\left(1-{\hat{b}}^{†}\hat{b}\right)}_{I}{\left({\hat{b}}^{†}\hat{b}\right)}_{J}\right\}. \end{array} \end{array}$$

Here $s\left(n\right):=\frac{1}{2}n\left(n-1\right)$ counts the number of anticommutators needed in ordering products like ${\hat{b}}_{I}^{†}{\hat{b}}_{I}$. For $I=\{i_{1},\ldots ,i_{k}\}$ with $i_{1}<i_{2}<\cdots <i_{k}$, we define ${\left({\hat{b}}^{†}\hat{b}\right)}_{I}:={\hat{b}}_{i_{1}}^{†}{\hat{b}}_{i_{1}}\ldots {\hat{b}}_{i_{k}}^{†}{\hat{b}}_{i_{k}}$, ${\left(1-{\hat{b}}^{†}\hat{b}\right)}_{I}:=\left(1-{\hat{b}}_{i_{1}}^{†}{\hat{b}}_{i_{1}}\right)\ldots \left(1-{\hat{b}}_{i_{k}}^{†}{\hat{b}}_{i_{k}}\right)$. As before, the commutator is symmetric in *I*, *J*, so the total diagonal contribution becomes

(A28) $$\frac{1}{2}{\left[\hat{S},\hat{V}\right]}_{\left(d\right)}=\sum_{IJ}{\left(-1\right)}^{s(|I|)+s(|J|)}\frac{{\left({\hat{b}}^{†}\hat{b}\right)}_{I}{\left(1-{\hat{b}}^{†}\hat{b}\right)}_{J}{\left|g_{IJ}\left({\hat{b}}^{†}\hat{b}\right)\right|}^{2}}{f(I=1,\;J=0)-f(I=0,\;J=1)}.$$

We end this appendix with an application to the Anderson impurity model. To simplify equations, we only consider the interaction of one conduction electron $\hat{b}$ and one localized orbital with two spin configurations possible, ${\hat{c}}_{+}$, ${\hat{c}}_{-}$. Under this simplification, the Hamiltonian reads

(A29) $$\hat{H}=\epsilon {\hat{b}}^{†}\hat{b}+\epsilon_{c}{\hat{c}}_{+}^{†}{\hat{c}}_{+}+\epsilon_{c}{\hat{c}}_{-}^{†}{\hat{c}}_{-}+U{\hat{c}}_{+}^{†}{\hat{c}}_{+}{\hat{c}}_{-}^{†}{\hat{c}}_{-}+\sum_{s=+,-}V{\hat{b}}^{†}{\hat{c}}_{s}+V^{*}\hat{b}{\hat{c}}_{s}^{†}.$$

*U* represents the Coulomb repulsion between the two electrons in the same orbital with opposite spin, and *V* represents the coupling between conduction electrons and the localized orbital. Using $\hat{b},{\hat{c}}_{+},{\hat{c}}_{-}$ as a bias ordering, we define functions

(A30) $$\begin{array}{cc} f(n,m,\ell ) & :=\epsilon n+\epsilon_{c}m+\epsilon_{c}\ell +Um\ell , \end{array}$$

(A31) $$\begin{array}{cc} g_{12}\left(n,m,\ell \right) & =g_{13}\left(n,m,\ell \right):=V. \end{array}$$

Then using our result Equation (A26), the transformation generator becomes

(A32) $$\begin{array}{cc} \hat{S} & ={\hat{b}}^{†}h_{12}{\hat{c}}_{+}+{\hat{b}}^{†}h_{13}{\hat{c}}_{-}-H.C. \end{array}$$

(A33) $$\begin{array}{cc} \; & =\sum_{s=+,-}{\hat{b}}^{†}\frac{V}{\left(\epsilon +\epsilon_{c}{\hat{c}}_{-s}^{†}{\hat{c}}_{-s}\right)-\left(\epsilon_{c}+\epsilon_{c}{\hat{c}}_{-s}^{†}{\hat{c}}_{-s}+U{\hat{c}}_{-s}^{†}{\hat{c}}_{-s}\right)}{\hat{c}}_{s}-H.C. \end{array}$$

(A34) $$\begin{array}{cc} \; & =\sum_{s=+,-}{\hat{b}}^{†}V\left\{\frac{1}{\epsilon -\epsilon_{c}}+\left(\frac{1}{\epsilon -\epsilon_{c}+U}-\frac{1}{\epsilon -\epsilon_{c}}\right){\hat{c}}_{-s}^{†}{\hat{c}}_{-s}\right\}{\hat{c}}_{s}-H.C., \end{array}$$

which is the form obtained in Ref. [15].
